# Long Term Amperometric Recordings in the Brain Extracellular Fluid of Freely Moving Immunocompromised NOD SCID Mice

**DOI:** 10.3390/s17020419

**Published:** 2017-02-22

**Authors:** Caroline H. Reid, Niall J. Finnerty

**Affiliations:** Chemistry Department, Maynooth University, Maynooth, County Kildare W23 F2H6, Ireland; caroline.reid@nuim.ie

**Keywords:** amperometry, nitric oxide, oxygen, real-time, characterization, NOD SCID, immunocompromised

## Abstract

We describe the in vivo characterization of microamperometric sensors for the real-time monitoring of nitric oxide (NO) and oxygen (O_2_) in the striatum of immunocompromised NOD SCID mice. The latter strain has been utilized routinely in the establishment of humanized models of disease e.g., Parkinson’s disease. NOD SCID mice were implanted with highly sensitive and selective NO and O_2_ sensors that have been previously characterized both in vitro and in freely moving rats. Animals were systemically administered compounds that perturbed the amperometric current and confirmed sensor performance. Furthermore, the stability of the amperometric current was investigated and 24 h recordings examined. Saline injections caused transient changes in both currents that were not significant from baseline. l-NAME caused significant decreases in NO (*p* < 0.05) and O_2_ (*p* < 0.001) currents compared to saline. l-Arginine produced a significant increase (*p* < 0.001) in NO current, and chloral hydrate and Diamox (acetazolamide) caused significant increases in O_2_ signal (*p* < 0.01) compared against saline. The stability of both currents were confirmed over an eight-day period and analysis of 24-h recordings identified diurnal variations in both signals. These findings confirm the efficacy of the amperometric sensors to perform continuous and reliable recordings in immunocompromised mice.

## 1. Introduction

The discovery of severe combined immunodeficiency (SCID) mice was a pivotal advancement in the development of immunodeficient mice for xenotransplantation [1,2]. The generation of these so called “humanized mice” that are reconstructed with human cells can facilitate analysis of the underlying mechanisms of human disease pathogenesis [1]. Heretofore a myriad of humanized mouse models exists using these mice specifically, due to their unparalleled ability to facilitate xenografts implantation and permit anatomical integration of cells and tissue. In particular models for cancer [3,4], HIV [5,6] and liver disease [7,8] have been established that facilitate focused research of human disease which was previously impossible in immunocompetent animals [1].

More recently, colleagues have generated and characterized a humanized mouse model of Parkinson’s disease (PD) through xenotransplantation of induced pluripotent stem cell (iPSC) derived dopaminergic neurons into NOD SCID mouse striatum [9,10,11]. These PD patient-derived iPSC’s are differentiated into dopaminergic neurons and transplanted into the striatum of NOD SCID mice to facilitate anatomical integration over several months. To date, there has been limited translation from existing animal models of PD to clinical neuroprotection in human populations. A large number of potentially neuroprotective compounds from a broad range of pharmacological groups have been identified in rodent and primate models, however, none have proven neuroprotective during clinical testing [12]. The general consensus is that this disparity is mainly due to the aetiopathogenic diversity of PD and humanised models can potentially bridge the gap between standard pre-clinical animal models of PD and clinical translation.

Neurochemical analysis has incorporated a wide range of techniques to date, in an attempt to elucidate the function and pathways of the various neurotransmitter systems in the intact mammalian brain. This includes the non-invasive techniques; positron emission topography (PET) and functional magnetic resonance imaging (fMRI), which monitor shifts in brain function [13] and neuronal activity [14] but suffer from poor temporal resolution. In comparison, invasive techniques, such as microdialysis and in vivo amperometry provide improved temporal resolution with the latter facilitating the real time recording of neurochemical dynamics in brain extracellular fluid (ECF) using highly sensitive and selective microelectrodes (sensors). This is achieved through the application of a suitable potential profile to the implanted sensor and the trend in a specific neurochemical is measured continuously and with sub second time resolution. Selectivity is critical due to the large number of possible interfering species present at relatively high concentrations, (e.g., ascorbic acid (AA), uric acid (UA), and neurotransmitters including dopamine (DA) and serotonin (5-HT)) in the mammalian brain. We have extensive experience in the development and characterization of in vivo sensors and heretofore long term amperometric recordings have been performed by most groups exclusively in freely moving rats [15,16,17,18,19,20]. However, the option to undertake measurements in freely moving immunocompromised mice offers access to novel humanized animal models of disease.

Therefore, the principle objective of the work described within was to characterize existing amperometric sensors for the continuous measurement of nitric oxide (NO) and oxygen (O_2_) and demonstrate their functionality in the striatum of freely moving NOD SCID mice. Moreover, the ability of this mice strain to facilitate chronic amperometric recordings was investigated thoroughly as a prerequisite for eventual deployment in a humanised mouse model of PD. This humanised mouse model will eventually facilitate unprecedented access to perform amperometric recordings within the microenvironment of transplanted PD human cells. Characterization of the respective sensor signals was achieved by validating the response of the sensor to the analyte of interest and exhibiting a stable signal over a number of days to facilitate reliable, continuous long term recordings. To the best of our knowledge, this article is the first to detail the continuous real time recording of NO and O_2_ levels in the brain of NOD SCID mice using amperometric sensors.

## 2. Materials and Methods

### 2.1. Chemicals and Solutions

Nafion^®^ (5 wt % solution in a mixture of lower aliphatic alcohols and H_2_O), graphite powder, and silicon oil, used in the manufacture of sensors were purchased from Sigma Aldrich Chemical Co. (Dublin, Ireland). The reagents used in phosphate buffered saline (PBS); sodium chloride (NaCl, 0.15 M), sodium hydroxide (NaOH, 0.04 M) and sodium hydrogen phosphate (NaH_2_PO_4_, 0.04 M) were purchased from Sigma Aldrich Chemical Co. For carbon monoxide (CO) and hydrogen sulphide (H_2_S) interference studies, CO gas (research grade) was sourced from BOC (Bluebell, Dublin, Ireland) and sodium sulphide nonahydrate was sourced from Sigma Aldrich Chemical Co.

### 2.2. In Vivo Compounds

All compounds used throughout the experiments were purchased from Sigma Chemical Co. (Dublin, Ireland) except for chloral hydrate which was obtained from BDH Laboratory Supplies (Poole, UK). In all cases, systemic administration of l-arginine (200 mg·kg^−1^), l-N^G^-Nitroarginine methyl ester hydrochloride (l-NAME, 30 mg·kg^−1^), chloral hydrate (350 mg·kg^−1^) and acetazolamide (Diamox, 50 mg·kg^−1^) occurred by intraperitoneal (i.p.) injection. All solutions were prepared using 0.9% saline solution and administered in a volume of 1 mL·kg^−1^.

### 2.3. Amperometric Sensor Recordings

Amperometric NO recordings were performed using previously characterized Nafion^®^-coated platinum (Pt) disk electrodes manufactured from Teflon^®^-insulated platinum/iridium (Pt/Ir 90%/10%) wire (127 µm bare diameter 5T, Science Products GmbH, Hofheim, Germany). The electrodes were modified as previously described [19,21,22]. NO pre-calibrations were performed in a standard three-electrode glass electrochemical cell containing PBS electrolyte. A saturated calomel electrode (SCE) acted as the reference electrode and a Pt rod utilised as the auxiliary electrode. Increasing concentrations of NO, manufactured in house [21], were injected in to the cell and the electrolyte solution was agitated using a magnetic stirrer (see Supplementary Material Figure S1). The current was then measured under quiescent conditions with an N_2_ atmosphere maintained over the solution to avoid quenching by ambient O_2._ Reported concentration changes are based on in vitro pre-calibration curves (average slope/sensitivity of 1040 ± 50 pA·µM^−1^, *n* = 23) recorded for all NO sensors (see Figure S1). NO sensor selectivity has been published previously [22] and performance was confirmed by determining the current responses to a myriad of electroactive interferents present in the brain extracellular fluid at concentrations representative of their physiological levels. The NO sensors demonstrated negligible responses (<1%) from most of the interfering species including ascorbic acid, serotonin, DOPAC, dopamine, l-gluthathione, hydrogen peroxide, 5-HIAA, homovanillic acid, nitrite and uric acid. Further selectivity studies were undertaken against more recent identified interferents including the electroactive gasotransmitters CO and H_2_S. Concentrations representative of their physiological levels were chosen [23,24]. A comparable selectivity over H_2_S (<1%, 1.6 ± 0.1 pA·µM^−1^, *n* = 4) and a slightly higher contribution from CO (ca. 2%, 22.7 ± 0.1 pA·µM^−1^, *n* = 4) was observed (see Figure S2). Previously published data [19,22] has reported on the stability and sensocompatability of the NO sensor. Membrane biofouling is a process that starts immediately upon contact of the sensor with the brain tissue. Previously we have shown in vitro that initial exposure of the sensor to proteins and lipids results in a significant decrease in sensitivity over the initial 24 h, however, an additional 48-h exposure had no further effect [22]. This resulted in a decrease of ca. 38% which is in line with other reports where a decrease of between 20% and 50% have been observed following initial exposure of sensors to brain tissue [25,26]. Furthermore, in vivo investigations support our assumption by confirming no significant difference in mean baseline current over a successive eight-day period in the striatum of freely moving rats [19]. The surface integrity of the implanted NO sensor has been validated further by systemic administration of electroactive interferents which caused no change in amperometric current in freely moving rats [19,20]. Prior to implantation of NO sensors, selectivity was confirmed by calibrating against ascorbic acid (AA) at a supraphysiological concentration which confirms membrane integrity (see Figure S3).

Amperometric O_2_ recordings were performed using carbon paste electrodes (CPEs). CPEs were manufactured from Teflon^®^-insulated silver (Ag) wire (200 µm bare diameter 8T, Advent Research Materials; Oxford, UK) using protocols previously characterized by others [18,27]. O_2_ pre-calibrations were performed in a standard three-electrode glass electrochemical cell containing PBS electrolyte. A saturated calomel electrode (SCE) acted as the reference electrode and a Pt rod utilised as the auxiliary electrode. As reported previously by others, the PBS was saturated with either N_2_ gas (0 µM O_2_, BOC Ireland, Dublin, Ireland), atmospheric air (240 µM O_2_, RENA air-pump) or O_2_ gas (1200 µM O_2_, BOC Ireland) for 20 min and the appropriate gaseous atmosphere was maintained for 15 min over the cell solution during quiescent recordings (see Figure S4). CPEs demonstrate excellent stability by retaining their structural integrity following exposure to in vivo fouling conditions. Reported concentration changes are based on in vitro pre-calibration curves (average slope/sensitivity of −1.69 ± 0.04 nA·µM^−1^, *n* = 17) (see Figure S4). The selectivity of CPEs has been reported previously by Bolger et al. [27] with negligible responses observed for the myriad of interferents investigated. Briefly, <1% contribution to the O_2_ signal was observed for ascorbic acid, homovanillic acid, l-gluthathione, l-cysteine, uric acid, serotonin, l-trypthophan, d-hydroascorbic acid, l-tyrosine, dopamine, DOPAC and 5HIAA. The absence of O_2_ signal interference was expected since the large faradaic currents generated as a result of O_2_ detection and the reductive nature of the applied potential mitigate against any potential sources of interference. Furthermore, it is widely documented that CPEs are extremely stable, even after a couple of weeks of continuous recording in the brain [28,29] due to the presence of the pasting oil in the CPEs affording them their resistance to poisoning in a lipid-protein matrix.

### 2.4. In Vivo Implantation and Surgery Protocol

NOD SCID mice (25–40 g) were housed in individually ventilated cages, with a maximum of five per cage in a temperature (17–23 °C), humidity and light controlled (12 h light, 12 h dark cycle) environment. Food and water were available *ad libitum*. Amperometric sensors were implanted following a previously described procedure [19,20] into the right or left striatum (*n* = 1/animal). Coordinates with the skull levelled between bregma and lambda were as follows; NO and O_2_ sensors: A/P + 0.5, M/L ± 1.5 from bregma and D/V − 4.0 from skull. Reference and auxiliary electrodes (8T Ag wires, 200 µm bare diameter) were placed in the cortex and soldered to a stainless steel support screw respectively. The electrodes were fixed to the skull with support screws and dental acrylate (Agnthos, Lidingö, Sweden). The mice were anesthetized with isoflurane anaesthesia (Abbott Laboratories, Dublin, Ireland), placed in a Stoelting stereotaxic frame (Stoeling Co., Wood Dale, IL, USA) and kept on a thermal pad with rectal probe (Stoeling Co.) to prevent hypothermia. A 10 mg·kg^−1^ s.c injection of the NSAID analgesic, Carprofen^®^ is administered at least 10 min prior to the incision in the scalp. Subsequently, Lidocaine analgesic (5 mg·kg^−1^) is applied topically to the incision site for local pain relief. Once surgery is complete, the animal is allowed to recover for a minimum of 24 h in its recording cage where it remains for the duration of the experiment. All experimental procedures were performed under license AE19124/P010 in accordance with the European Communities Regulations 2002 (Irish Statutory Instrument 165/2013).

### 2.5. Animal Experimental Conditions

All amperometric recordings in freely moving mice were carried out with the animal in an open top recording cage. Implanted amperometric electrodes were connected to a potentiostat through a six-pin Teflon socket and a bespoke screened four core cable manufactured in house which was mounted through a swivel slip ring (CPC Farnell, Preston, UK) above the subject’s head. This cable and swivel arrangement allowed free movement of the subject and permitted continuous amperometric recordings for up to five days at a time with an intermittent two-day disconnection period. The desired potentials: +900 mV vs. Ag wire, and −650 mV vs. Ag wire were applied to the NO and O_2_ sensors respectively and the currents were allowed to stabilise for a minimum of 24 h prior to any perturbation being performed.

### 2.6. Instrumentation, Software and Data Analysis

The amperometric current was detected using a potentiostat (Quadstat, eDAQ Ltd., Sydney, Australia) and converted using an A/D converter (eCorder, eDAQ Ltd.). The digital signal was then recorded using eChart software (v5.5, eDAQ Ltd.) running on a Dell Laptop. Signal processing was performed on collected data to further improve signal to noise ratios using the eChart software. All data analysis was performed using GraphPad Prism v5 (GraphPad Software Inc., San Diego, CA, USA). For characterization and stability investigations, all figures presented are averaged across all animals and had baseline currents normalised to 100% illustrating the overall change in current as a % of the pre-perturbation baseline level. This removes both inter electrode and inter animal variability by ensuring that the presented current changes are representative of the data from all the animals used in each study. For 24-h investigations, all figures presented had currents converted to concentration using in vitro calibration data and a 30 min baseline was averaged for each sensor which was then set as zero. The significance of differences observed was estimated using the Student’s *t*-test for paired/unpaired observations (where appropriate) and one-way ANOVAs with Bonferroni post hoc test (where appropriate). Two-tailed levels of significance were used with *p* < 0.05 considered to be significant. All data is presented as mean ± standard error (SEM), with *n* = number of animals except for 24 h recordings where *n* = number of days or nights/number of animals. One sensor implanted per animal.

## 3. Results and Discussion

### 3.1. Effect of Control Saline Injections on NO and O_2_ Currents in the Striatum of Freely Moving NOD SCID Mice

**Figure 1:**
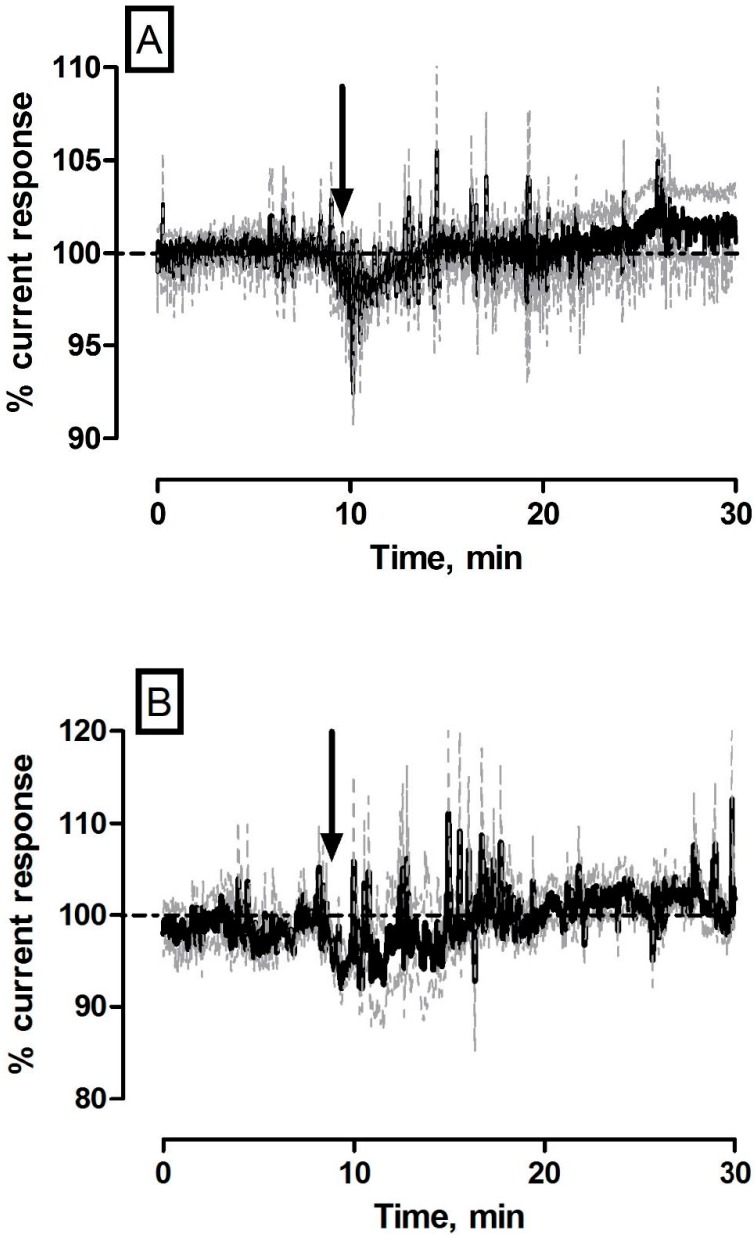
Averaged raw data % current response of (**A**) NO and (**B**) O_2_ sensors implanted in the striatum of NOD SCID mice (*n* = 5) to a 1 mL·kg^−1^ injection of 0.9% saline. Arrows indicate point of injection. Mean % current response represented by black trace, % error represented by grey trace.

All compounds were prepared using 0.9% saline solution and administered by intraperitoneal injection in a volume of 1 mL·kg^−1^. Therefore, it was imperative to characterize the sensor response to control saline injections. The averaged raw data % current responses following saline administration (*n* = 5) are detailed in Figure 1. Briefly, the maximum current change for NO (−4 ± 3 pA) and O_2_ (−2 ± 3 nA) sensors were not significantly different from pre-injection baseline levels (490 ± 79 pA (*p* = 0.30) and 199 ± 14 nA (*p* = 0.64)) respectively. Maximum responses were recorded for NO after (1.0 ± 0.1 min) and O_2_ (3.0 ± 1.7 min) that corresponded to percentage changes of (NO, −0.4% ± 0.8% and O_2_, −0.6% ± 1.3%) and concentration changes of (NO, 4 ± 3 nM and O_2_, 1 ± 2 µM) based on pre-calibration data. The currents had returned back to a new recorded level for NO (488 ± 80 pA, *p* = 0.45) and O_2_ (200 ± 14 nA, *p* = 0.49) after 3.0 ± 0.9 min and 10.8 ± 7.1 min respectively.

These transient responses are in line with previous findings from data obtained from brain extracellular fluid in freely moving rats. Short lived changes from baseline levels were reported for both NO [19,20] and O_2_ [18] sensors following saline injection with no long lasting effect recorded in either signal.

### 3.2. Effect of Systemic Administration of the NOS Inhibitor l-NAME on NO and O_2_ Currents in the Striatum of Freely Moving NOD SCID Mice

**Figure 2:**
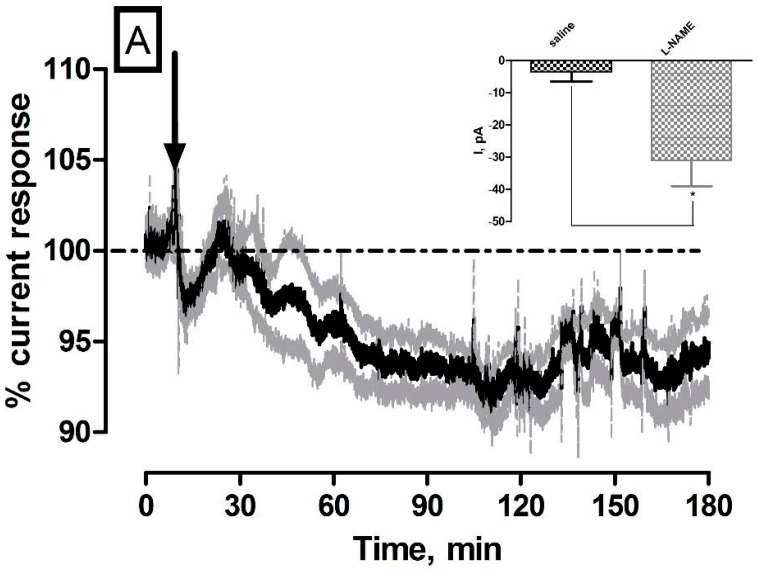

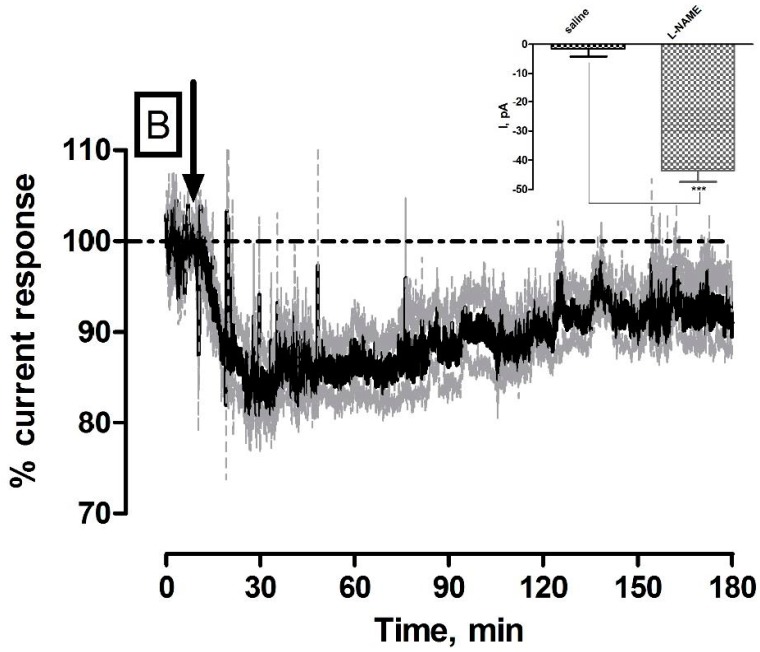
Averaged raw data % current response of (**A**) NO and (**B**) O_2_ sensors implanted in the striatum of NOD SCID mice to a 30 mg·kg^−1^ injection of l-NAME (*n* = 5). Arrows indicate point of injection. Mean % current response represented by black trace, % error represented by grey trace. Insets: Comparison of max current response (ΔI) for saline control vs. l-NAME ((**A**) NO sensor (*p* < 0.05), unpaired *t*-test (*n* = 5) and (**B**) O_2_ sensor (*p* < 0.001), unpaired *t*-test (*n* = 5)). Data represented as ΔI ± SEM as compared to baseline. * denotes level of significance.

l-NAME is a non-selective nitric oxide synthase (NOS) inhibitor which exerts its function through direct competition with the pre cursor L-arginine for its binding site on the NOS enzyme [30]. NOS inhibition has been shown to induce constriction of cerebral vessels and decrease tissue blood flow in the brain providing evidence that basal NO is involved in the maintenance of cerebral perfusion [31,32,33]. The averaged raw data % current traces following the systemic administration of 30 mg·kg^−1^ l-NAME (*n* = 5) are illustrated in Figure 2. The maximum current change for NO (−31 ± 8 pA) and O_2_ (−44 ± 4 nA) sensors were significantly different from pre-injection baseline levels (436 ± 86 pA (*p* < 0.01) and 199 ± 12 nA (*p* < 0.001) respectively. Furthermore, significant changes against saline controls were observed for both NO (*p* < 0.05, see Figure 2A inset) and O_2_ (*p* < 0.001, see Figure 2B inset). Maximum responses were recorded for NO (41 ± 18 min) and O_2_ (28 ± 6 min) that corresponded to percentage changes of (NO, −6% ± 1% and O_2_, −19% ± 3%) and concentration changes of (NO, 30 ± 8 nM and O_2_, 26 ± 2 µM) based on pre-calibration data. The currents had reached a new recorded level for NO (430 ± 84 pA, *p* = 0.17) and O_2_ (181 ± 12 nA, *p* < 0.01) after 180 min. These results corroborate previously reported findings whereby l-NAME has been utilised in the characterization of the NO sensor in wistar rats to great effect [19,20]. Heretofore, its application in O_2_ sensor characterization studies has not been reported. Notwithstanding this, it is an ideal compound for characterization studies since the tissue O_2_ current measured by the amperometric sensor is determined by the balance between vasculature supply and cellular utilisation. By this rationale, the decrease observed in Figure 2B can be attributed to a decrease in O_2_ supply arising from vasoconstriction and utilization remaining constant. A net decrease in O_2_ current is thus recorded. Taken collectively, a decrease in both NO and O_2_ current over a 180 min support a correlatory response and thus provide direct real time evidence of NO’s involvement in cerebral circulation. However, a slight discrepancy in maximum response times is observed which could be explained by the non-selective nature of l-NAME. Specifically, inhibition of endothelial NOS (eNOS), which is localised to the smooth muscle of blood vessels, would exert its effect directly on blood flow by inducing vasoconstriction which would reduce the levels of O_2_ available in the brain extracellular fluid. Moreover, it is a generally accepted phenomenon that neuronal NOS (nNOS) is the most abundantly present isoform in the brain that is tightly associated with NMDA receptor function on post synaptic membranes [34].

Considering this, it is therefore plausible that the majority of NO measured at the sensor source is of neuronal form which would explain the temporal disparity observed between both signals in Figure 2.

### 3.3. Effect of Systemic Administration of the NO Precursor l-arginine on NO Currents in the Striatum of Freely Moving NOD SCID Mice

NO production proceeds through the amino acid l-arginine, which in the presence of various co-factors and molecular O_2,_ binds to its site on the NOS enzyme to produce NO and l-citrulline:

$$L-arginine+2NADPH+O_{2}\to L-citrulline+NO+2NADP^{+}$$

**Figure 3:**
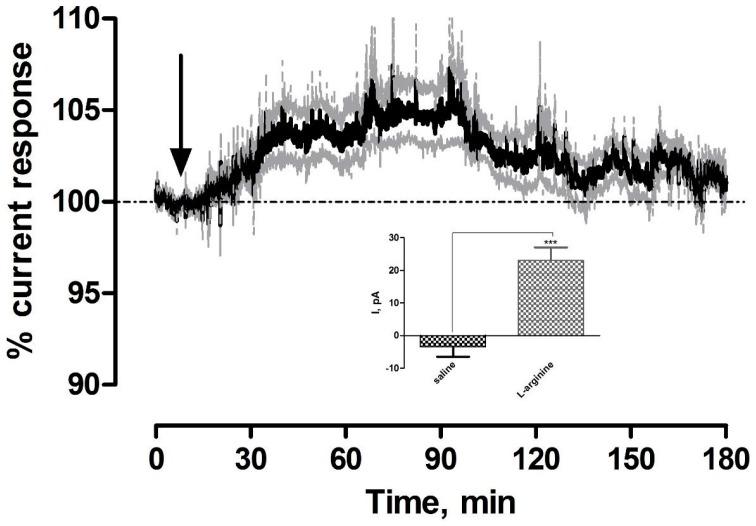
Averaged raw data % current response of NO sensors implanted in the striatum of NOD SCID mice to a 200 mg·kg^−1^ injection of l-arginine (*n* = 5). Arrow indicates point of injection. Mean % current response represented by black trace, % error represented by grey trace. Inset: Comparison of ΔI for saline control vs. l-arginine (*p* < 0.001), unpaired *t*-test (*n* = 5). Data represented as ΔI ± SEM as compared to baseline. * denotes level of significance

The nitrogen of NO is derived from the guanidine nitrogen atoms of l-arginine and the oxygen from molecular O_2_ with the production of a stoichiometric equivalent concentration of the co-product l-citrulline [35]. l-arginine has been used previously to characterize the NO sensors in freely moving rats with significant differences observed against baseline and control saline administrations [20,22]. Furthermore, local perfusion of the precursor using retro-microdialysis initiated significant increases in NO current [19]. The averaged raw data trace following the systemic administration of 200 mg·kg^−1^ l-arginine is illustrated in Figure 3. A significant increase (*p* < 0.001) in current was observed (23 ± 4 pA, *n* = 5) in comparison to pre-injection baseline levels of 545 ± 99 pA and against saline controls (*p* < 0.001, see Figure 3 inset). This current change reached a maximum level after 48 ± 9 min and corresponded to a percentage change of 5% ± 1% and concentration change of 22 ± 4 nM based on pre-calibration data. A new baseline current of 545 ± 98 pA was reached after 128 ± 18 min and was not significantly different (*p* = 0.95) when compared against initial pre-injection levels. These results confirm that amperometric currents increase following systemic administration of the pre-cursor in NO synthesis, l-arginine, supporting the efficacy of the sensor in determining increasing NO concentrations.

### 3.4. Effect of Systemic Administration of Chloral Hydrate Anaesthetic on O_2_ Currents in the Striatum of Freely Moving NOD SCID Mice

**Figure 4:**
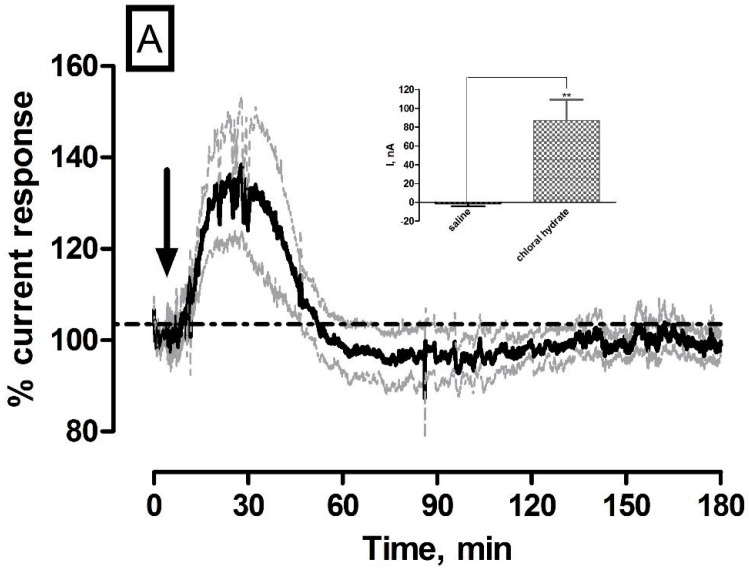

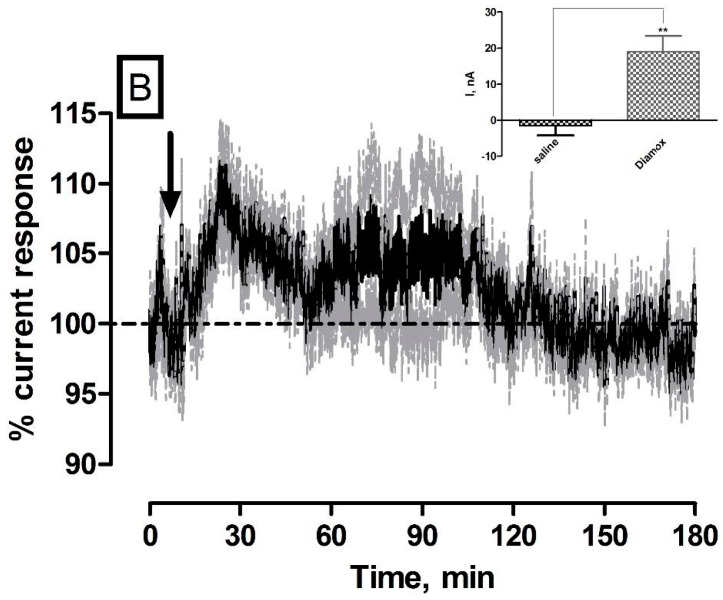
Averaged raw data % current response of O_2_ sensors implanted in the striatum of NOD SCID mice to a (**A**) 350 mg·kg^−1^ injection of chloral hydrate (*n* = 5) and (**B**) 50 mg·kg^−1^ injection of Diamox. Arrows indicate point of injection. Mean % current response represented by black trace, % error represented by grey trace. Insets: Comparison of ∆I for (**A**) saline control vs. chloral hydrate (*p* < 0.01), unpaired *t*-test (*n* = 5) and (**B**) saline control vs. Diamox (*p* < 0.01), unpaired *t*-test (*n* = 5). Data represented as ∆I ± SEM as compared to baseline. * denotes level of significance.

Systemic administration of the non-volatile anaesthetic chloral hydrate was utilised to investigate its effect on striatal O_2_. The anaesthetic response has been previously characterized using the amperometric O_2_ sensors in freely moving rats with a long lasting increase in current recorded over a number of hours [18]. It causes general CNS depression by rapidly metabolising into trichloroethanol and trichloroacetic acid. The chloral hydrate effect on suppressing neuronal activity is quick, resulting in a large increase in O_2_ current due to the supply of O_2_ from the vasculature exceeding its utilisation. The averaged raw data % current trace following the systemic administration of 350 mg·kg^−1^ chloral hydrate is illustrated in Figure 4A. A significant increase (*p* < 0.05) in current was observed (87 ± 22 nA, *n* = 5) in comparison to pre-injection baseline levels of 201 ± 10 nA and against saline controls (*p* < 0.01, see Figure 4A inset). This current change reached a maximum level after 13 ± 2 min and corresponded to % change of 40% ± 15% and concentration change of 51 ± 13 µM based on pre-calibration data.

A new baseline current of 195 ± 13 nA was reached after 97 ± 9 min and was not significantly different (*p* = 0.33) when compared against initial pre-injection levels. These results strongly corroborate previously reported findings from O_2_ sensors implanted in wistar rats [18] and confirmed the efficacy of the amperometric sensor at measuring increases in O_2_ concentration in the striatum of NOD SCID mice.

### 3.5. Effect of Systemic Administration of the Carbonic Anhydrase Inhibitor Diamox on O_2_ Currents in the Striatum of Freely Moving NOD SCID Mice

The carbonic anhydrase inhibitor acetazolamide (Diamox) has been shown to increase cerebral blood flow (CBF) and O_2_ levels in both humans and animal models [36,37]. Measurement of the CBF or blood velocity response to Diamox is commonly used to assess the vasodilatory capacity of the cerebral circulation and moreover, it has been routinely utilised in the characterization of the amperometric O_2_ sensor in Wistar rats [18]. The averaged raw data % current response following 50 mg·kg^−1^ Diamox administration (*n* = 5) is detailed in Figure 4B. Briefly, the maximum current change (19 ± 4 nA) was significantly different from pre-injection baseline levels (221 ± 23 nA (*p* < 0.05) and against saline controls (*p* < 0.01, see Figure 4B inset). A maximum response was recorded after 15 ± 3 min that corresponded to a percentage change of 8% ± 2% and a concentration change of 11 ± 2 µM based on precalibration data. The currents had returned back to a new recorded level (220 ± 23 nA, *p* = 0.80) after 97 ± 27 min. These results strongly corroborate previously reported findings from sensors implanted in wistar rats [18] and confirm the efficacy of the O_2_ sensor at measuring concentration changes in the striatum of NOD SCID mice.

### 3.6. Stability of the NO and O_2_ Currents in the Striatum of Freely Moving NOD SCID Mice

**Figure 5:**
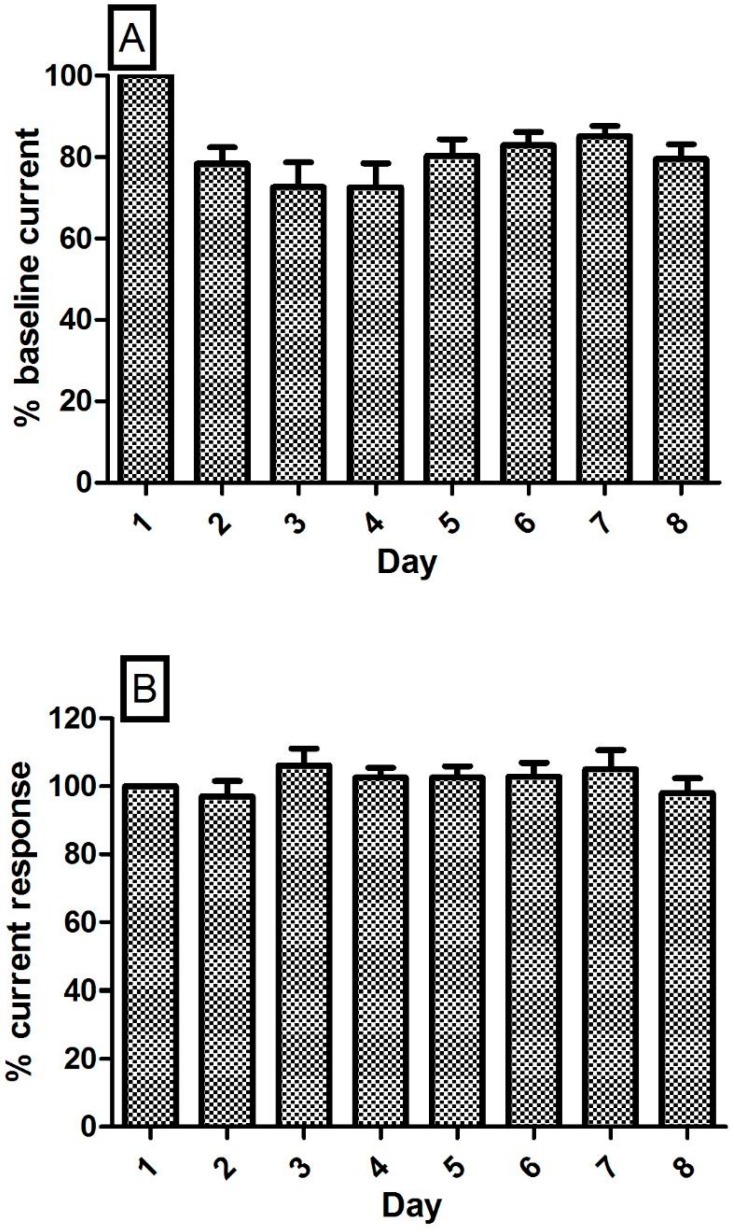
Average % baseline currents for (**A**) NO sensors (*n* = 6) and (**B**) O_2_ sensors (*n* = 5) implanted in the striatum of NOD SCID mice.

The mammalian brain presents a complex chemical environment that includes electrode poisons such as lipids and proteins and a tissue matrix that both restricts mass transport to the electrode surface and reacts physiologically to the presence of the sensor. It is imperative that the sensor demonstrates efficient stability for continuous long term recordings. The potential benefits of pre-treating microelectrodes with lipids [29,38] and proteins [39] has been investigated by research groups to permit more accurate pre-calibration techniques by mitigating this sensitivity decrease observed upon implantation. These studies have established that exposing the electrodes to fouling agents in vitro may serve as a good model of the behaviour of implanted sensors in brain tissue. Previous in vivo investigations using the described NO and O_2_ sensors in the brain ECF of freely moving rats, confirm no significant difference in mean baseline NO current over a successive eight day period [19] or in the mean baseline O_2_ current over a number of weeks [27]. This follows an early reduction in baseline stability which has been corroborated by other groups where a decrease in sensitivity has been observed following initial exposure of sensors to brain tissue [25,26]. A similar phenomenon was observed in the striatum of NOD SCID mice. Figure 5 illustrates the average % baseline current recorded from the mice over an eight-day period which supports previous evidence recorded from Wistar rats. Day 1 baselines were normalised to 100% and all subsequent baselines are normalised to a percentage of the baseline recorded on Day 1.

Initially there was a drop in current observed on Day 2 which has been reported by other groups, however, no significant variation in NO (*p* = 0.62, one-way ANOVA; six animals) and O_2_ (*p* = 0.74, one-way ANOVA; five animals) signal was reported over subsequent days which demonstrates the stability of both amprometric sensors over an eight-day recording period.

### 3.7. 24 Hours Recording of NO and O_2_ Concentration Dynamics in the Striatum of Freely Moving NOD SCID Mice

**Figure 6:**
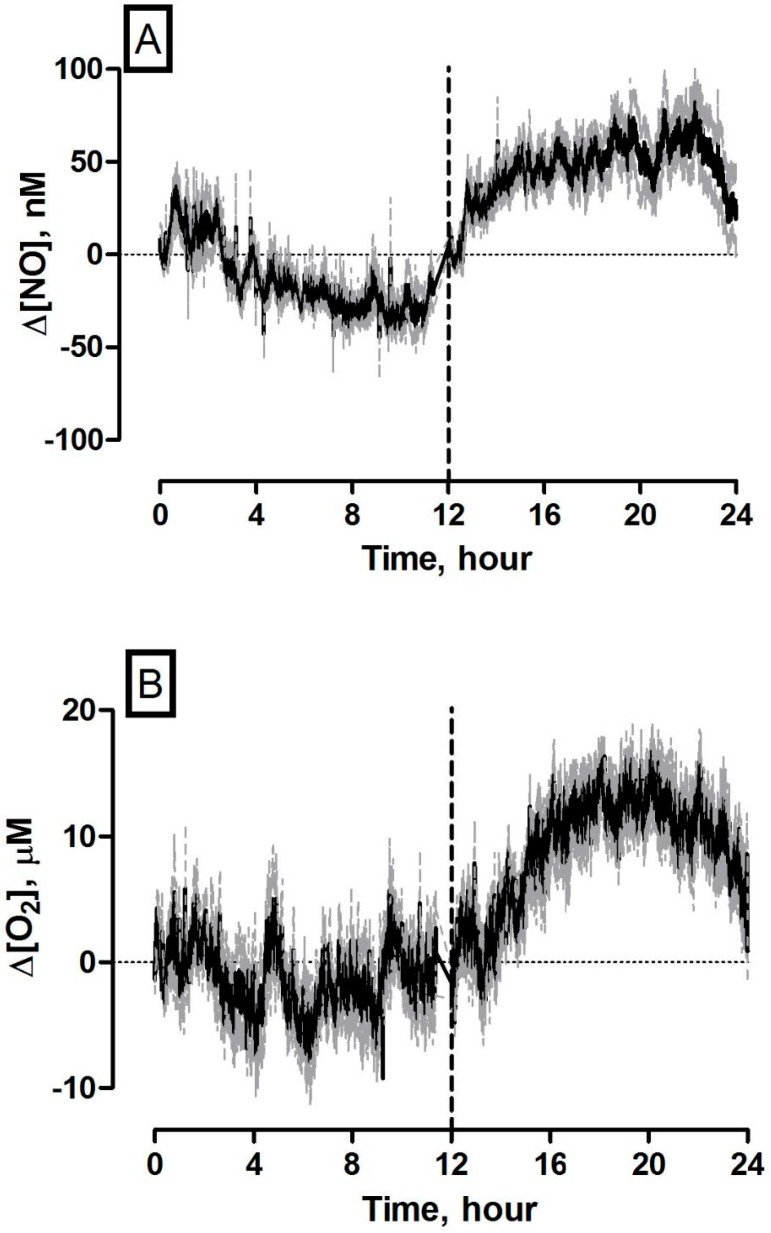
Averaged 24 h concentration dynamics measured using (**A**) NO and (**B**) O_2_ sensors implanted in the striatum of NOD SCID mice. Mean concentration represented by black trace, error represented by grey trace. 0–12 h represent light phase (07.00–19.00) and 12–24 h represent dark phase (19.00–07.00). Vertical dashed line signifies end of light phase/start of dark phase.

Continuous 24 h amperometric recordings were examined to identify approximate concentration changes over extended periods of inactivity (light cycle) and increased locomotor activity (night cycle) based on pre-calibration data. It is apparent from Figure 6 that increasing concentrations of NO (*n* = 12/5) and O_2_ (*n* = 16/5) coincide with increasing locomotor and behavioural activity routinely associated with nocturnal species.

In contrast, light phase concentrations fluctuated around basal levels during extended periods of hypolocomotion and inactivity (NO: *n* = 11/5 and O_2_: *n* = 10/5). These findings support a diurnal modulation of both gaseous molecules in NOD SCID mouse striatum that corresponds to changes in behavioural and general locomotor activity. The obvious similarities observed in NO and O_2_ trends during nocturnal periods (see Figure S5) may represent increases in cerebral haemodynamics associated with elevated neural activity during the dark phase. Previously, Endo et al. reported a comparable diurnal variation in local CBFin awake rat hippocampus, over a 24 h period using the hydrogen clearance technique [40]. They proposed that this increase is directly related to increased neuronal function. The close relationship between neuronal activity and local regulation of CBF through intricate vasoactive mechanisms has long been recognised [41]. Nevertheless, the mechanisms that modulate the coupling between CBF and neuronal activity in the mammalian brain remain unresolved. Traditionally, it was accepted that active neurons generate a metabolic signal in response to increasing O_2_ or glucose demand, triggering an increase in CBF. In recent times a theory has evolved suggesting that neurotransmitter mediated signalling, particularly by glutamate has a critical function in regulating CBF through the initiation of a multifaceted vasodilatory pathway [42], including NO. The temporal effects of NO and O_2_ currents illustrated in Figure 6 and Figure S5 support the latter mechanism. Increasing NO concentrations precede O_2_ increases during periods of neuronal activation that is closely associated with nocturnal activity to compensate for cerebral metabolic demands. Separate work undertaken by Kostin and colleagues using the amperometric NO sensor in freely moving rats, corroborate this prolonged increase measured during the dark phase [43]. However, given the hypothesised role of NO in the regulation of sleep, their approach demonstrated that NO levels in the perifornical-lateral hypothalmic area exhibit sleep-wake as well as diurnal modulation with highest levels measured during the dark phase. They postulate that NO concentrations in this brain region are increased during the dark phase to inhibit neurons and aid in the promotion of sleep. Further work is necessary to determine the exact mechanisms responsible, however, the diurnal variations observed during these 24-h recordings using the amperometric sensors are a clear indication of their capability to measure long term neurochemical dynamics in awake, freely moving subjects.

## 4. Conclusions

The principle objective of the work described within was to demonstrate the functionality of previously characterized amperometric sensors for the continuous real time monitoring of NO and O_2_ in the striatum of freely moving NOD SCID mice. The latter animal strain is utilsed routinely for humanized models of disease and the reported findings within are critical for the future deployment of these amperometric sensors in humanized mouse models of disease. Table 1 summarizes the characterization data obtained for both NO and O_2_ sensors from the brain extracellular fluid of the immune-compromised mice.

Briefly, transient current changes were observed for both sensors following control saline injections. Significant differences in NO current were recorded for both l-NAME and l-arginine relative to baseline and saline injections. Similarly, significant differences in O_2_ current were recorded for l-NAME, chloral hydrate and Diamox with respect to baseline and saline administrations. The stability of the NO and O_2_ current was confirmed over an eight-day period and analysis of 24-h signal recordings identified diurnal variations for both sensors. The latter confirms the efficacy of the amperometric technique at measuring long-term neurochemical variations in immuno-compromised mice.

## Figures and Tables

**Table 1:** Summary of in vivo characterization data for NO and O_2_ sensors implanted in the striatum of freely moving NOD SCID mice (*n* = 5). * denotes level of significance.

Administration	∆ I	∆ Conc	vs. Baseline	vs. Saline
Saline (NO)	−4 ± 3 pA	4 ± 3 nM	*p* = 0.30	-
Saline (O_2_)	−2 ± 3 nA	1 ± 2 µM	*p* = 0.64	-
l-NAME (NO)	−31 ± 8 pA	30 ± 8 nM	** *p* < 0.01	* *p* < 0.05
l-NAME (O_2_)	−44 ± 4 nA	26 ± 2 µM	*** *p* < 0.001	*** *p* < 0.001
l-arginine (NO)	23 ± 4 pA	22 ± 4 nM	*** *p* < 0.001	*** *p* < 0.001
Chloral hydrate (O_2_)	87 ± 22 nA	51 ± 13 µM	* *p* < 0.05	** *p* < 0.01
Diamox (O_2_)	19 ± 4 nA	11 ± 2 µM	* *p* < 0.05	** *p* < 0.01

## Supplementary Materials

The following are available online at http://www.mdpi.com/1424-8220/17/2/419/s1.
