# Supplementary material for: Long Term Amperometric Recordings in the Brain Extracellular Fluid of Freely Moving Immunocompromised NOD SCID Mice
Source: Sensors (Basel). 2017 Feb 22;17(2):419. doi: 10.3390/s17020419 (PMC5335951; doi:10.3390/s17020419)
Supplement: Supplementary file 1 [file sensors-17-00419-s001.pdf]

Supplementary Material

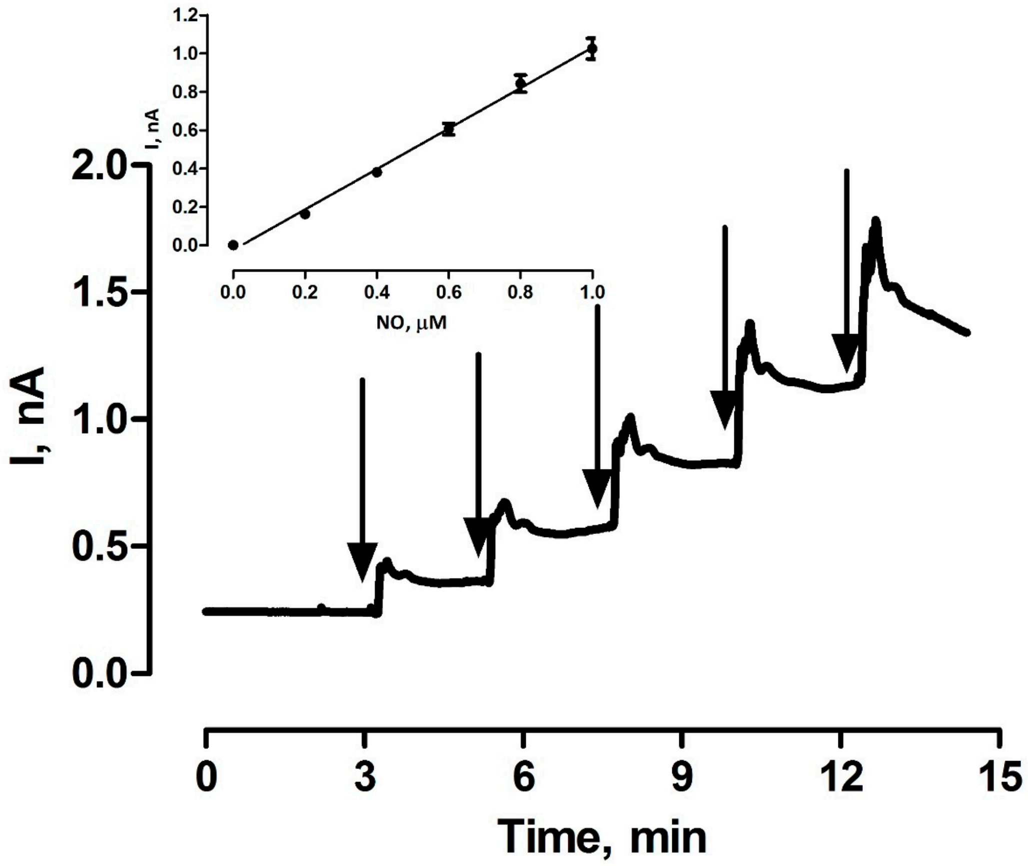

Figure S1: Typical *in vitro* data obtained for a 0 to 1 μM NO calibration using Nafion® coated Pt disk sensors. Arrows indicate addition of 0.2 μM NO aliquots. *Inset*: Current concentration profile for NO calibration on Nafion® coated Pt disk sensors (*n* =23), r^2 = 0.99. All concentration profile data is presented as mean ± SEM.

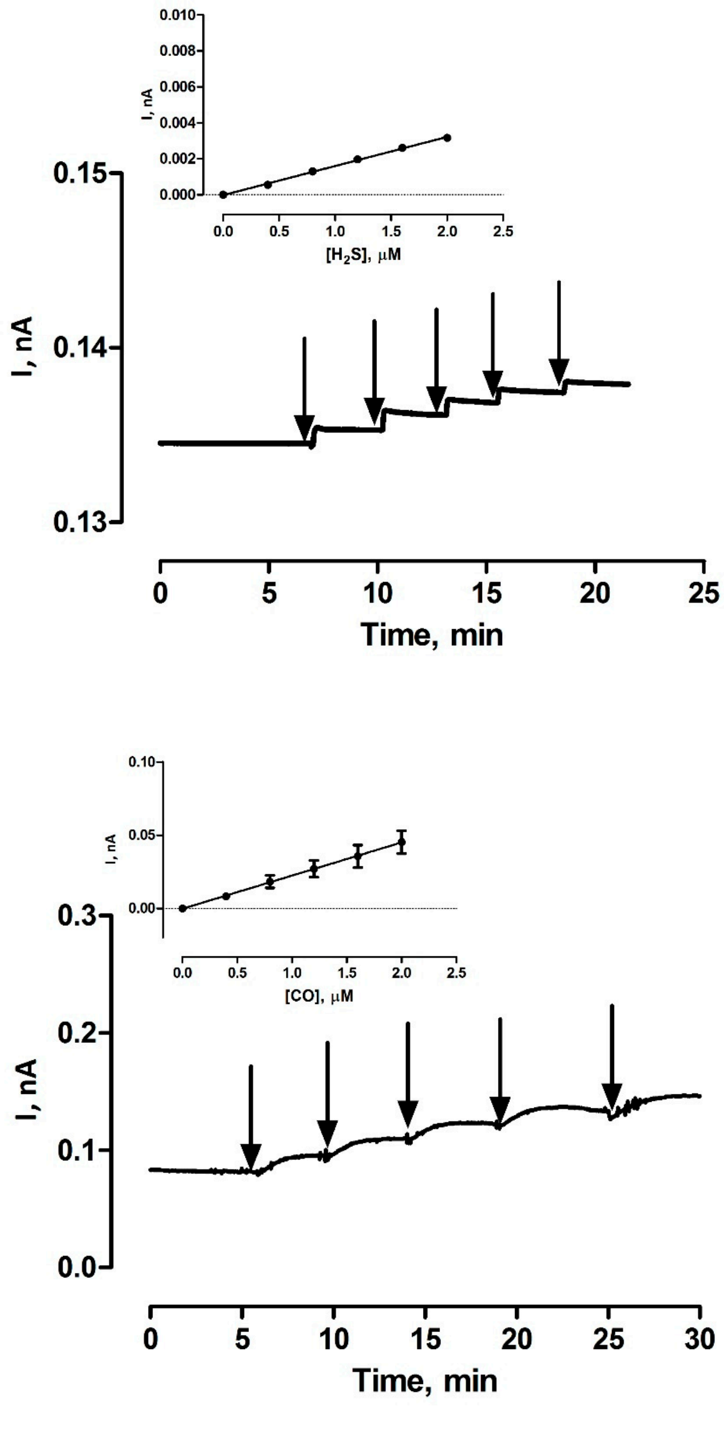

Figure S2: **Typical *in vitro* data obtained for (top, main) 0 to 2 μM H_2S and (bottom, main) 0 to 2 μM CO calibrations on Nafion® coated Pt disk NO sensors. Arrows indicate addition of 0.4 μM aliquots. *Inset*: Current concentration profile for (top) H_2S and (bottom) CO calibration on Nafion® coated Pt disk NO sensors (*n* =4), r^2 = 0.99. All concentration profile data is presented as mean ± SEM.**

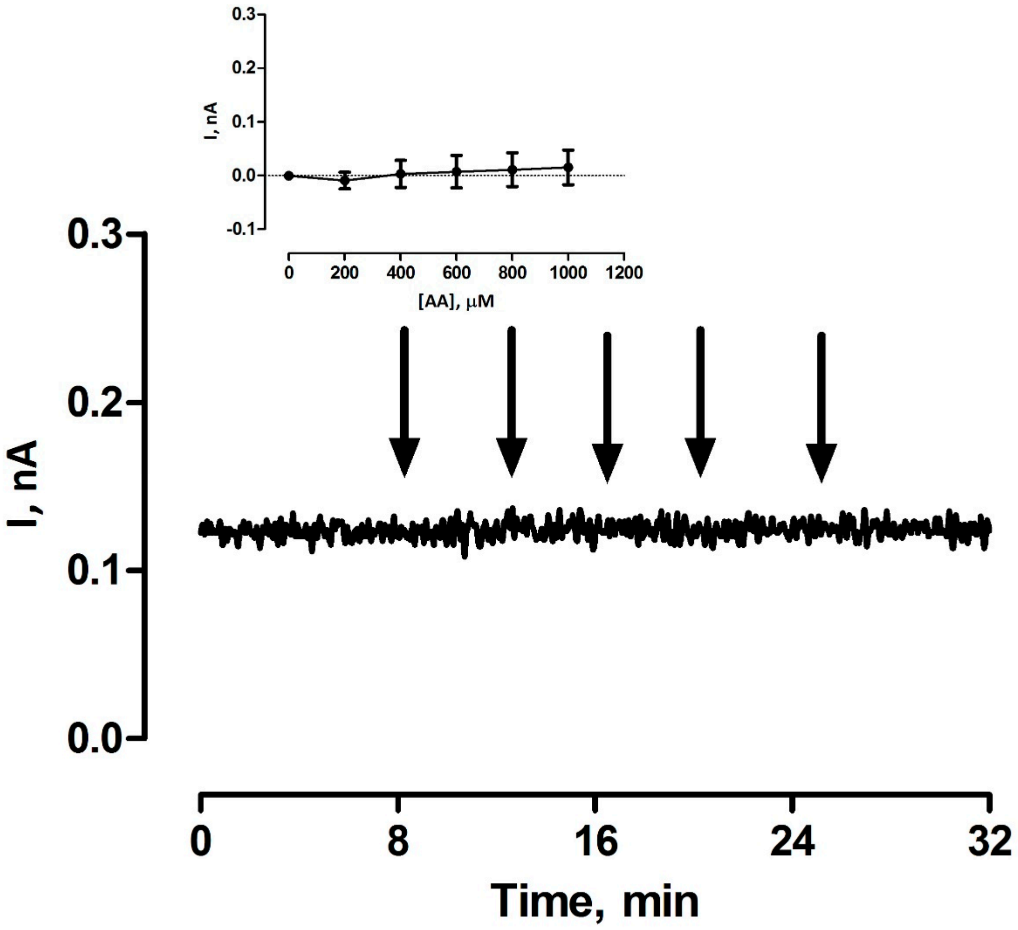

Figure S3: Typical *in vitro* data obtained for a 0 to 1000 μM AA calibration using Nafion® coated Pt disk NO sensors. Arrows indicate addition of 200 μM AA aliquots. *Inset*: Current concentration profile for AA calibration on Nafion® coated Pt disk NO sensors (*n* =17), r^2 = 0.99. All concentration profile data is presented as mean ± SEM.

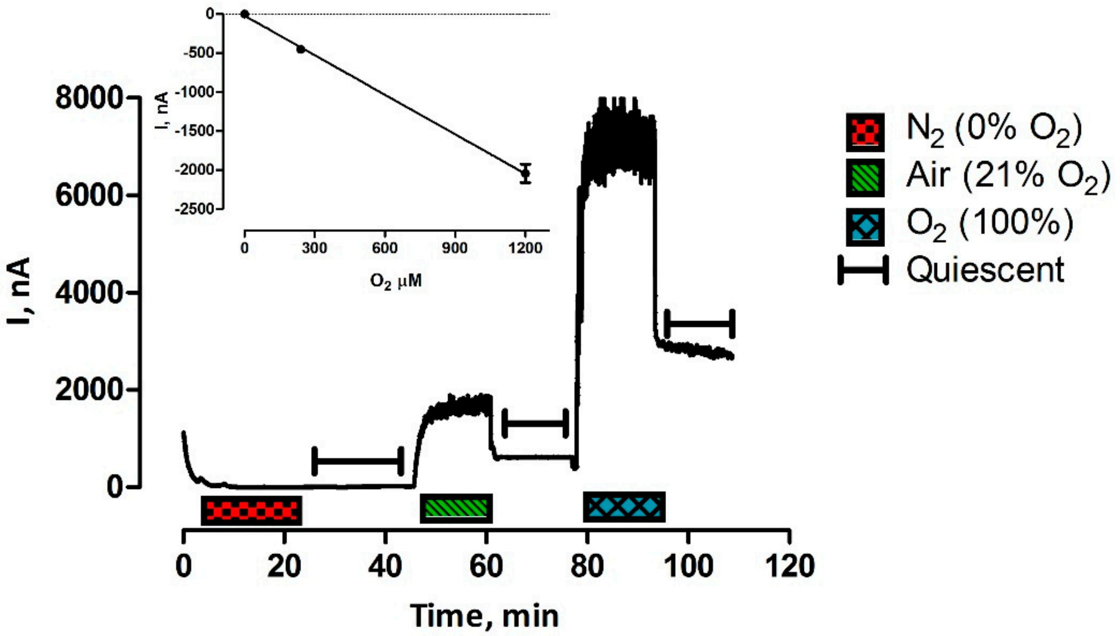

Figure S4: Typical *in vitro* data obtained for a 0 to 1200 μM O_2 calibration using CPE. *Inset*: Current concentration profile for O_2 calibration on CPEs (*n* =17), r^2 = 0.99. All concentration profile data is presented as mean ± SEM.

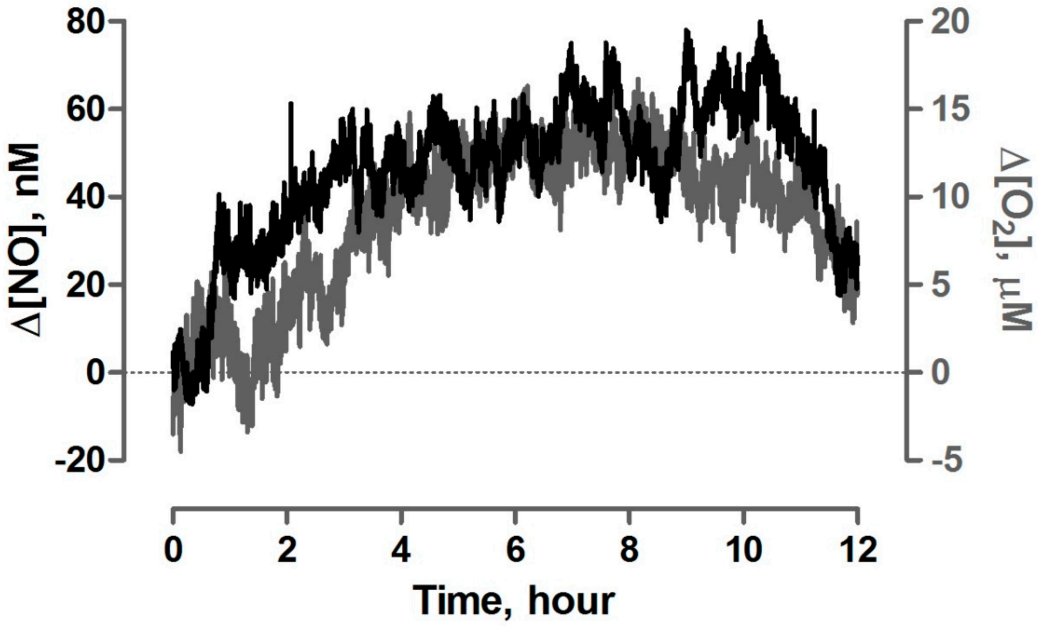

Figure S5: Comparison of averaged dark phase (19.00 – 07.00) concentration dynamics measured using NO (black trace, left y-axis) and O_2 (grey trace, right y-axis) sensors implanted in the striatum of NOD SCID mice.
